# Evaluation of high-throughput isomiR identification tools: illuminating the early isomiRome of *Tribolium castaneum*

**DOI:** 10.1186/s12859-017-1772-z

**Published:** 2017-08-03

**Authors:** Daniel Amsel, Andreas Vilcinskas, André Billion

**Affiliations:** 1 Fraunhofer Institute for Molecular Biology and Applied Ecology, Department of Bioresources, Winchester Str. 2, 35394 Giessen, Germany; 2 Institute for Insect Biotechnology, Heinrich-Buff-Ring 26-32, 35392 Giessen, Germany

**Keywords:** Insectomics, microRNA, Small RNA sequencing, isomiRID, isomiR-SEA, Miraligner

## Abstract

**Background:**

MicroRNAs carry out post-transcriptional gene regulation in animals by binding to the 3' untranslated regions of mRNAs, causing their degradation or translational repression. MicroRNAs influence many biological functions, and dysregulation can therefore disrupt development or even cause death. High-throughput sequencing and the mining of animal small RNA data has shown that microRNA genes can yield differentially expressed isoforms, known as isomiRs. Such isoforms are particularly relevant during early development, and the extension or truncation of the 5' end can change the profile of mRNA targets compared to the original mature sequence. We used the publicly available small RNA dataset of the model beetle *Tribolium castaneum* to create the first comparative isomiRome of early developmental stages in this species. Standard microRNA analysis software does not specifically account for isomiRs. We therefore carried out the first comparative evaluation of the specialized tools isomiRID, isomiR-SEA and miraligner, which can be downloaded for local use and can handle next generation sequencing data.

**Results:**

We compared the performance of isomiRID, isomiR-SEA and miraligner using simulated Illumina HiSeq2000 and MiSeq data to test the impact of technical errors. We also created artificial microRNA isoforms to determine the effect of biological variants on the performance of each algorithm. We found that isomiRID achieved the best true positive rate among the three algorithms, but only accounted for one mutation at a time. In contrast, miraligner reported all variations simultaneously but with 78% sensitivity, yielding isomiRs with 3' or 5' deletions. Finally, isomiR-SEA achieved a sensitivity of 25–33% when the seed region was mutated or partly deleted, but was the only tool that could accommodate more than one mismatch. Using the best tool, we performed a complete isomiRome analysis of the early developmental stages of *T. castaneum*.

**Conclusions:**

Our findings will help researchers to select the most suitable isomiR analysis tools for their experiments. We confirmed the dynamic expression of 3′ non-template isomiRs and expanded the isomiRome by all known isomiR modifications during the early development of *T. castaneum*.

**Electronic supplementary material:**

The online version of this article (doi:10.1186/s12859-017-1772-z) contains supplementary material, which is available to authorized users.

## Background

MicroRNAs (miRNAs) are post-transcriptional regulators of gene expression that influence a wide range of biological processes [1]. In insects, the dysregulation of miRNA expression during metamorphosis is often lethal [2–4]. Mature miRNAs are ~22 nucleotides in length and the 3′ end binds to a member of the Argonaute protein family to form an RNA-induced silencing complex (RISC) [5]. The RISC binds target mRNAs within the 3′ untranslated region (UTR) or in the coding sequence via complementary base pairing with the miRNA seed region (nucleotides 1–8) and in some cases also the compensatory region (nucleotides 13–16) [6]. RISC binding inhibits further processing of the mRNA, thus blocking translation or promoting degradation [1].

The biogenesis of miRNAs can involve the production of isoforms known as isomiRs [7]. These are thought to be produced deliberately as separate products with defined roles in the cell, and do not represent errors of transcription or errors of sequencing [8]. The isomiRs may be extended or truncated at either end compared to the mature miRNA, presumably due to imperfect cleavage by Drosha or Dicer [9]. Recent studies indicate that 5′ isomiRs undergo a seed region shift which changes the set of target mRNAs compared to the original miRNA [10]. The set of target mRNAs can also be changed by nucleotide editing [11, 12]. Mature miRNAs may also acquire non-templated polynucleotide 3′ tails generated by nucleotidyltransferases [13]. This phenomenon has been observed during early insect development as part of maternal transcriptome regulation [14, 15].

The results described above show that miRNAs and isomiRs play important roles during animal development, especially insect morphogenesis. To gain more insight into the prevalence of isomiRs in insects we screened the publicly available small RNA dataset of the model beetle *Tribolium castaneum* originally focusing exclusively on 3′ non-templated isomiRs in the early development stages [15]. The data had already undergone a conservative form of isomiR investigation by iteratively truncating the non-templated 3′ ends until a certain minimal length was reached or the sequence perfectly matched a known miRNA. We investigated the performance of tools for isomiR identification that account for more than non-templated 3′ tails. Several such tools have been developed but no comparative benchmarks are available. We selected a set of three candidate tools that are suitable for the analysis of high-throughput sequencing data and compared their performance to identify the best software. Using a simulated test set of Illumina reads and a set of artificial isomiRs, we investigated the influence of technical errors and biological variations on each type of software and determined the sensitivity and specificity for each case. From these values, we calculated a final weighted performance score for each tool. Taken individually, the two cases also provide detail information on the eventual need of post system error correction, considering the system error test case and possible detection leaks of isomiR types, uncovered by the biological variant test set.

## Methods

### IsomiR analysis software

Seven isomiR mining and alignment tools are currently available as non-proprietary software (Table 1). Three of them are command line tools that can be downloaded and integrated into high-throughput pipelines, and these are described in more detail below. We used these three methods for a comparative benchmark of their individual performance on simulated reads. If adjustable, we used the default settings in each tool without read abundance cutoffs. We wanted each tool to utilize its entire search space and therefore did not set the parameters to a common minimum in the case of mismatches, additions and deletions.

**Table 1:** List of non-proprietary isomiR alignment programs

Program	Usage	Alignment method	Publisher
isomiR-SEA 1.60	Command line isomiR-SEA_1_6 -s tca -l 10 -b 4 -i < in_path > −p < out_path > −ss 6 -h 11 -m < mature_mir_file > −t < countfile>	User-defined seed size (default 6)	Urgese et al. [21]
isomiRID 0.53	Command line standard config file	bowtie1	de Oliveira et al. [22]
miraligner 3. Feb 2016	Command line java -jar miraligner.jar -sub 1 -trim 3 -add 3 -s tca -freq	8 nt seed	Pantano et al. [23]
IsomiRage	Desktop GUI	bowtie1	Muller et al. [24]
DeAnnIso	Webapp	bowtie1 and BLAST	Zhang et al. [25]
isomiRex	Webapp	bowtie1	Sablok et al. [26]
miR-isomiRExp	Webapp – offline	bowtie1	Guo et al. [27]

The three command line tools were used for our comparative evaluation. The others were discarded because they were incompatible with local high-throughput pipelines

#### isomiR-SEA

The C++ program isomiR-SEA focuses on the seed region of miRNAs. It is a standalone executable file without dependencies and can be run with parameters in the command line. It requires the mature miRNA file from miRBase and the sequence reads. The reads must be collapsed and reformatted with the unique read and its abundance in one line. The algorithm extracts the seed regions from the mature miRNAs and groups them together. At first, the reads are screened for seed regions. When found, the seed region is extended without gaps in both directions and the correct position of the seed block is checked. The algorithm continues the extension towards the 3′ end and allows a second mismatch if the distance between the two mismatches falls within a user-defined threshold. The alignment is then extended further until either the third mismatch or the end of the read is encountered. Then the scores for each aligned read are computed. The output files are grouped into unique mapping reads, ambiguous reads that map more than once, and ambiguous selected reads that also map to various miRNAs but can be assigned to a unique one due to an internal scoring function (Table 2). There are also “unique”, “ambiguous” and “ambiguous selected” output files, referring to the miRNA instead of the read.

**Table 2:** Result files generated by isomiR-SEA

Unique	Tag_unique
Unique_ambigue_selected	
Ambigue	Tag_ambigue
Ambigue_ambigue_selected	Tag_ambigue_selected

The *tag* files focus on the read, whereas the others report the variants of the miRNA

#### isomiRID

The Python 2.7 script isomiRID uses bowtie [16] to map small RNA sequencing reads against reference precursor miRNAs. The script uses a configuration file in which the user can specify the paths of the executables, the data and the parameters. In the first round, perfect matches against the precursors are identified. An optional filtering step of the unaligned reads against the corresponding transcriptome or genome can be performed to filter reads not from miRNAs. In the second step, reads with one mismatch are taken into account. Iterative trimming of the 5′ and 3′ ends is used to seek potential non-templated miRNA isoforms. The findings are filtered according to user-defined abundance cutoffs and the results are concatenated into output files, allowing for reads with more than one mapping location. The output is a tab separated file in which every mapped read is aligned under the assigned precursor sequence together with the identified type of isoform and the abundance of the read.

#### Miraligner

The Java tool miraligner, originally from the SeqBuster package but now independent, is a single jar file without dependencies. It uses a collapsed read file and the miRNA hairpin FASTA file from miRBase [17] together with the hairpin secondary structure file. The reads are mapped to the hairpin sequences via seeds of eight nucleotides, allowing one mismatch within the sequence. It allows up to three non-templated nucleotide additions at the 3′ end, as well as up to three nucleotides that differ from the mature 3′ or 5′ ends. This allows a slight shift of the precursor compared to the annotated position in the hairpin secondary structure file from miRBase. We used the default settings with a maximum substitution of one and a trimming/adding of three. The output is a tab separated file. It shows a result for each mutation type, the read sequence together with the number of its assignments, as well as the names of the miRNA.

### Technical error simulation

We evaluated the effect of Illumina sequencing errors on the accuracy of isomiR identification by each tool. The small RNA sequencing data were simulated using ART [18] (version Mount Rainier 2016–06-05) with the Illumina HiSeq2000 and MiSeq-v1 sequencing system in single-strand mode: art_illumina -c 1000 -ss [HS20|MSv1] -i < pattern_file_with_miR_length_X > −l < miR_length_X > −o < output>. We grouped all miRNAs with the same length into one file and ran the command for each file separately. Afterwards, the files were merged into one. These sequencing systems are widely used for small RNA sequencing and mirror the most recently analyzed biological data. To ensure traceability, the simulated sequences must be uniquely assignable to their source. In case of isomiRID and miraligner, this can be achieved by the sequence header. The results of isomiR-SEA lack this header and a traceability can only be provided by sequence identity. Therefore, we had to ensure a uniqueness of miRNAs and their reads. We used the 430 *T. castaneum* mature miRNAs from miRBase v21 and merged identical sequences. This new set of 422 sequences was then used as the pattern for the two simulations, with a coverage of 1000 reads per sequence. Due to the nature of the simulation program, about half of the 422,000 reads were sequenced as a reverse complement and were therefore omitted from further analysis. The remaining reads, 210,753 for HiSeq2000 and 210,961 for MiSeq-v1, were then filtered for redundancy. This resulted in 13,850 unique reads for HiSeq2000 and 5964 unique reads for MiSeq-v1. This ensured a coverage of 14–32 read variants per original miRNA and therefore a broad variety of technical errors. The correct assignment of erroneous reads to its source was treated as true positive, because the tools cannot distinguish between error and mutation. An additional analysis after the identification step might be of use, depending on the investigation.

### Biological variation simulation

**Fig. 1:**
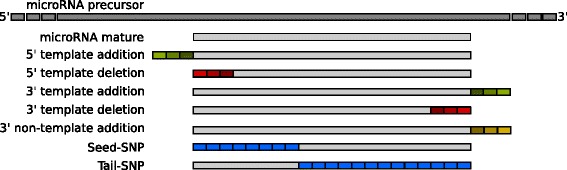
The seven types of isomiR custom mutations. The green boxes represent nucleotide additions. The red boxes represent nucleotide deletions. The yellow boxes represent non-template additions. The *blue boxes* show the positions of SNPs

In order to evaluate the isomiR programs comprehensively using biological data, we created custom sequences based on the mature *T. castaneum* miRNAs from miRBase v21. This mirrored seven different types of isoforms (Fig. 1). Both the 5′ and 3′ template isoforms were divided into truncated and extended variants. For the truncated variants, we created three different 5′ and three different 3′ isomiRs per mature microRNA, by iteratively trimming one nucleotide from the 5′ or from the 3′ end respectively. For the three 5′ and three 3′ extended variants, we added one nucleotide to the particular end of the mature miRNA, using the precursor miRNA as the template, until a maximum of three additions was reached. The 12 3′ non-templated isoforms per mature miRNA were created by adding one nucleotide of the same type to the mature miRNA, until a total of three nucleotides were added. We divided the single nucleotide polymorphism (SNP) isoforms into two distinct classes: the seed-SNPs and the tail-SNPs. We replaced each nucleotide from position 1 to 8 with the remaining three nucleotides for the seed-SNPs dataset and from position 9 to the end for the tail-SNPs dataset, resulting in three SNP isoforms per miRNA nucleotide position. This allowed us to distinguish the performance of seed-based search algorithms between seed and tail SNPs. We again kept the created reads non-redundant to ensure the traceability of the mapped reads by sequence identity. Our resulting test set finally mirrored each possible variation and therefore provided a general unbiased condition.

### Performance evaluation

We evaluated each algorithm using the simulated technical and biological *T. castaneum* reads. The results were classified as true positives (TP), false positives (FP) and false negatives (FN). True negatives (TN) were excluded because they were not needed for further calculations. Correctly assigned reads were treated as true positives. A wrongly assigned read was treated as false positive and a missing assignment to the correct miRNA was treated as false negative. We also calculated the sensitivity (TP/(TP + FN)) and the specificity (TP/(TP + FP)) of each isomiR software. Three possible approaches can be used to evaluate small RNA sequencing reads with more than one mapping location. One is to ignore multi-mapping reads completely and focus on distinct results. The second option is to group the miRNAs with the same read together. The third is to distribute the abundance of the read among the number of mapped miRNAs [19]. We decided to use the third approach because the other options would modify the isomiRome.

### *Tribolium castaneum* small RNA sequencing data

Recent studies have indicated the presence of abundant non-templated 3′ isomiRs during the early development stages of *T. castaneum* and *Drosophila melanogaster* [14, 15]. We used the publicly available *T. castaneum* small RNA sequencing data from the GSE63770 project (Table 3) for our analysis. Those datasets monitor the development of *T. castaneum* from the egg (including the switch from maternal to zygotic transcription after 5 h) until hatching (144 h) [15].

**Table 3:** List of publicly available *T. castaneum* small RNA datasets representing different developmental stages

ID	Sample	Transcription
GSM1556886	Oocyte small RNA replicate 1	Maternal
GSM1556887	Oocyte small RNA replicate 2	Maternal
GSM1556888	Embryo small RNA 0–5 h replicate 1	Maternal
GSM1556889	Embryo small RNA 0–5 h replicate 2	Maternal
GSM1556890	Embryo small RNA 8–16 h	Zygotic
GSM1556891	Embryo small RNA 16–20 h	Zygotic
GSM1556892	Embryo small RNA 20–24 h	Zygotic
GSM1556893	Embryo small RNA 24–34 h	Zygotic
GSM1556894	Embryo small RNA 34–48 h	Zygotic
GSM1556895	Embryo small RNA 48–144 h	Zygotic

After ~5 h, the maternal transcription phase ends and zygotic transcription commences [15]

### Adapter trimming and quality filter

The *T. castaneum* small RNA sequencing data was trimmed with cutadapt [20] v1.8.3, using -m 17 as the minimum read length, −M 30 as the maximum read length and --trim-n, to trim potential N characters at the ends of the reads. We excluded reads with at least one N character in their sequence.

## Results

We selected three high-throughput isomiR analysis tools suitable for command line use and investigated the effects of biological variation and sequencing-derived errors on the results produced by each tool (Additional file 1: Figure S1). The technical test sets were created with ART, using a copy rate of 1000 reads per miRNA. We additionally created biological test sets geared to known miRNA isoforms and again reduced them to a non-redundant set, allowing us to measure the effects of biological variation on the results produced by each tool. We finally generated scores for each tool and selected the appropriate software for the analysis of the *T. castaneum* isomiRome.

### Effect of technical errors on isomiR analysis

**Fig. 2:**
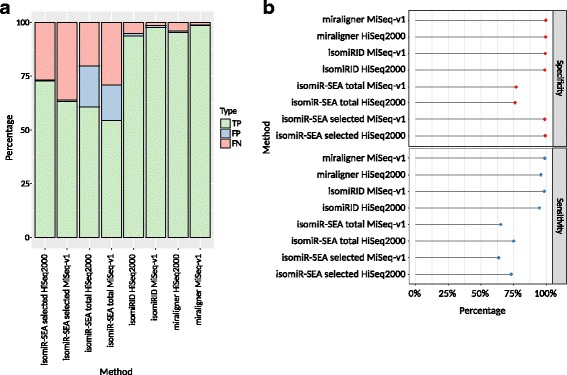
Technical error benchmarking of the isomiR analysis tools. Each algorithm was applied to the simulated sequencing error test set. (**a**) Plot of the true positive, false positive and false negative values from the mapping of erroneous reads against miRNAs. (**b**) Calculated sensitivity and specificity values

We created simulated HiSeq2000 and MiSeq-v1 reads based on mature miRNA templates from miRBase v21 with ART [18]. The multiple isomiR-SEA result files were divided into two distinct evaluations. We distinguished between the total results reported by isomiR-SEA (unique - reads that mapped only once and ambigue - reads that mapped more than once) on one hand and the selected results, already filtered by isomiR-SEA (unique - reads that mapped only once and ambigue_selected - reads that mapped more than once, but were disambiguated through isomiR-SEA internal scorings) on the other. The number of isomiR-SEA false positives was lower in the selected set compared to the total results, falling by more than 15% for MiSeq-v1 and more than 18% for HiSeq2000 (Fig. 2a). However, the false negative rate increased by nearly 7% for both HiSeq2000 and MiSeq-v1 in the selected set. This is also reflected in the increased specificity (+23.15% for HiSeq2000 and +21.97% for MiSeq-v1) and weaker sensitivity (−1.95% for HiSeq2000 and −1.37% for MiSeq-v1) (Fig. 2b). The results produced by miraligner and IsomiRID were almost identical for this benchmark: miraligner achieved ~1.60% and ~0.78% more true positives than IsomiRID for the HiSeq2000 and MiSeq-v1 data, respectively, ~0.50% fewer false positives for both HiSeq2000 and MiSeq-v1, as well as 1.13% and 0.21% fewer false negatives for HiSeq2000 and MiSeq-v1, respectively.

### Effect of biological variation on isomiR analysis

**Fig. 3:**
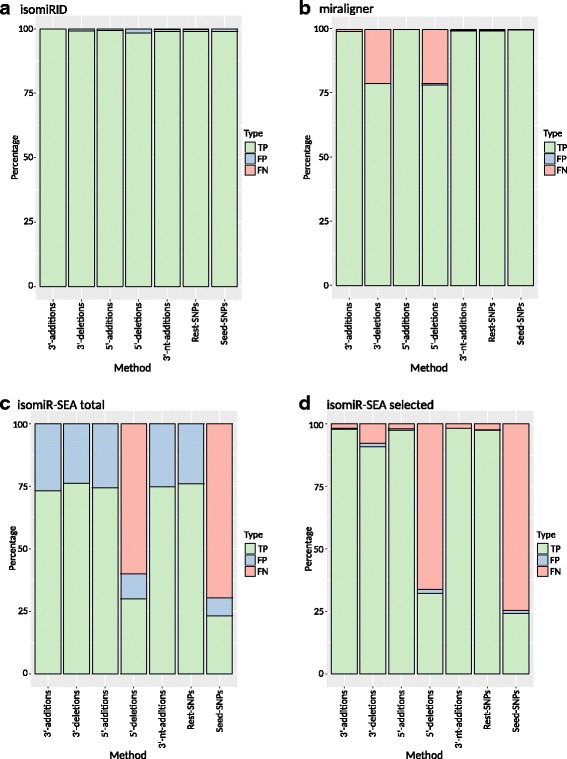
True positive, false positive and false negative results generated by isomiR analysis tools. The algorithms isomiRID (**a**), miraligner (**b**), isomiR-SEA total (**c**) and isomiR-SEA selected (**d**), were applied to the simulated biological variation test set

We tested the three tools for their ability to process artificially mutated miRNAs representing isomiR variations. Although isomiRID achieved a true positive rate of at least 98.4%, the false positive rate was 0.7–1.6% for every variant, except 3′ additions with 0.08% false positives (Fig. 3a). In contrast, miraligner achieved a true positive rate of >99.5% and a false negative rate of ≤0.5% for all variants except 3′ and 5′ deletions, where the false negative rate was ~21% (Fig. 3b). We again distinguished between total and selected isomiR-SEA results, attempting to eliminate multi-mapping reads. For the total results (Fig. 3c) we observed for nearly every type of mutation a false positive rate of ~25%, with the exception of seed-SNPs and 5′ deletions where the false positive rates ranged from ~7% to ~10%. We also observed false negative rates of 60% and 70% in these two variants. For the selected results (Fig. 3d) the false positive rate ranged from 0% for 3′ non-templated additions to 1.5% for 5′ deletions. The false negative rates for 3′ and 5′ template additions, 3′-non-templated additions and variants covering mutations outside the seed region were all approximately 2%. However, the false negative rate increased to 7.8% for 3′ truncations, 66% for 5′ truncations and 77% for seed-SNPs.

**Fig. 4:**
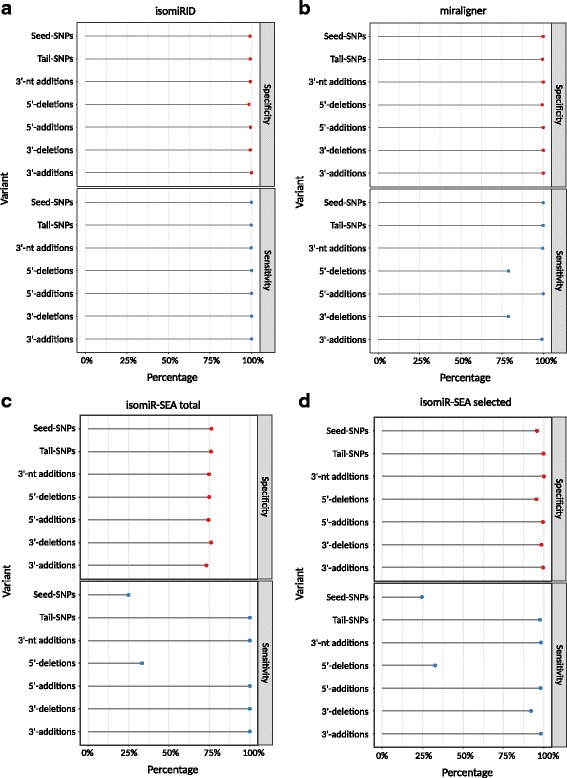
Sensitivity and specificity of the isomiR analysis tools isomiRID (**a**), miraligner (**b**), isomiR-SEA total (**c**) and isomiR-SEA selected (**d**). The values were calculated using the TP, FP and FN metrics from the analysis of the biological variation test set

The sensitivity of isomiRID was >99% for every variant and 100% for truncations and extensions at either end of the sequence (Fig. 4a). In contrast, the sensitivity of miraligner for deletion variants was 79% and ~99% for every other variant (Fig. 4a). When considering the total results, the sensitivity of isomiR-SEA was 100% for every variant except seed-SNPs and 5′ deletions, where the sensitivity fell to 33% and 25%, respectively (Fig. 4c). When considering the filtered results, the sensitivity of isomiR-SEA ranged from 92% to 98% for most variants but again showed a lower sensitivity for seed-SNPs and 5′ deletions, with values almost identical to the total results (Fig. 4d). The specificity of isomiRID ranged from 98% for 5′ truncations to 99% for 3′ templated additions (Fig. 4a). The specificity of miraligner was 100% for templated 3′ and 5′ additions and 3′ truncations, and 99% for 5′ truncations (Fig. 4b). The specificity of isomiR-SEA (total results) was 73–76% (Fig. 4c) whereas the selected results improved the specificity to 95–98% (Fig. 4d).

In order to exclude a possible influence of the read length to the result, we tested the effect of artificial read lengths on the method detection efficiency (Additional file 1: Figures S2 and S3). IsomiRID had a weak anti-correlation between read length and false positive rate of −0.36. Its highest false negative rate was at the length of 18 nt. Miraligner had a moderate anti-correlation between read length and false negative rate of −0.53. This was mainly caused by read lengths between 15 and 17 nt. The two variations of isomiR-SEA performed equally, concerning the correlations. They show an anti-correlating value of −0.24 and −0.22 for false negatives, caused by read lengths between 18 and 26 nt.

### Overall performance scores for isomiR analysis software

**Fig. 5:**
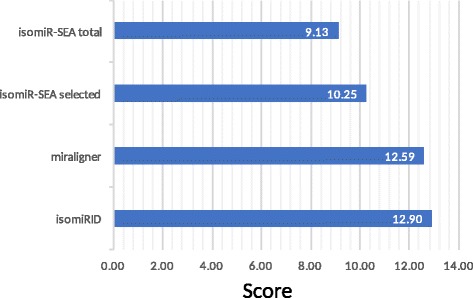
Overall ranking of the isomiR analysis tools. The points were calculated by weighting true positives, false positives and false negatives together with the impact on the seed region

Each of the analysis tools was scored according to its performance when handling technical errors and biological variations as described above, resulting in the overall ranking presented in Fig. 5. We calculated the f-scores for each tool and weighted them depending on their impact on real data. The highest score of 12.90 points was achieved by isomiRID, followed by miraligner with 12.59 points and isomiR-SEA with 9.13 and 10.25 points for the total and selected data, respectively.

We calculated the f-scores for each testing variant. Then each f-score was weighted regarding to its impact on the targeting mechanism of the miRNA isoform. We assigned a weighting of 1 to the templated 3′ additions and truncations as well as the tail-SNPs because these do not affect the seed region and therefore the range of mRNA targets is unchanged. However, variants that affect the seed region such as seed-SNPs and 5′ additions and truncations were weighted with a multiplier of 2, because changes in this region can modify the mRNA target range and are more biologically significant. We also assigned a multiplier of 2 to the 3′ non-templated additions because of their impact during early development. Finally, every score was summed up for each tool and set as final score for the evaluation.

In selecting a method for analysis of the *T. castaneum* isomiRome, we also considered aspects of general usability. For example, isomiRID uses precursor sequences and calculates a dot alignment for every matching read, but the number of dots is sometimes incorrect. This results in a visually shifted mature sequence alignment. Furthermore, isomiRID also reports only one mutation at a time and does not mark 5p and 3p miRNAs. In contrast, miraligner can report all isoforms simultaneously but replaces reads with the same name. We also observed that the precursors tca-miR-3811c-1 and tca-miR-3851a-1 were not reported in the test output even though they were provided in the input file, whereas the precursors tca-miR-3811c-2 and tca-miR-3851a-2 were present. We compared each pair and found that those precursors share the same mature sequence.

We nevertheless selected miraligner for the further analysis of the *T. castaneum* isomiRome, using the same settings as in the test cases. It scored 0.31 fewer points than isomiRID but 2.34 more than isomiR-SEA using the filtered data. It reported all variations for each read and generated fewer false positives than isomiRID, which reports only one mutation at a time and therefore cannot be used for comprehensive isomiRome profiling. Precursor overwriting was ignored because we focused on the mature sequences.

### The isomiRome of *Tribolium castaneum*

**Fig. 6:**
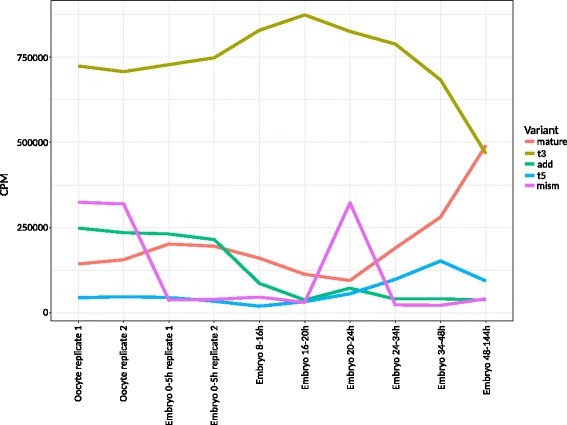
Counts per million reads per condition, normalized by the number of multi-mapping reads. This shows the 3' non-templated additions (add), the mature sequence (mature), the mismatches (mism), templated 3' additions and deletions (t3) and templated 5' additions and deletions (t5)

We calculated the number of reads that matched each type of isomiR variant in counts per million (CPM). The multi-mapping reads were normalized by the number of assigned microRNAs to avoid overrepresentation (Fig. 6). We observed an increase in the number of 3′ non-templated additions (add) during the maternal transcription phase (oocyte replicates 1 and 2, embryo 0–5 h replicates 1 and 2) which agreed with previous studies in *T. castaneum* [15] and *D. melanogaster* [14]. We also observed an initial increase in the number of templated 3′ additions (t3) peaking during the embryonic phase 16–20 h and declining thereafter. The mature sequences showed an opposing expression profile, with the lowest point at 16–20 h and an increase thereafter. The final phase had a higher CPM than the templated 3′ additions. The 5′ templated additions (t5) were present at constantly low levels with the exception of the 34–48 h phase. The SNP isoforms (mism) ranked second highest in expression value in the oocytes, which is even higher than previously reported for non-templated 3′ additions [15]. The expression of SNP isoforms dropped to one of the lowest values of all variants in the post-oocyte phases although there was a second significant peak during the 20–24 h phase before falling to minimal levels thereafter.

**Fig. 7:**
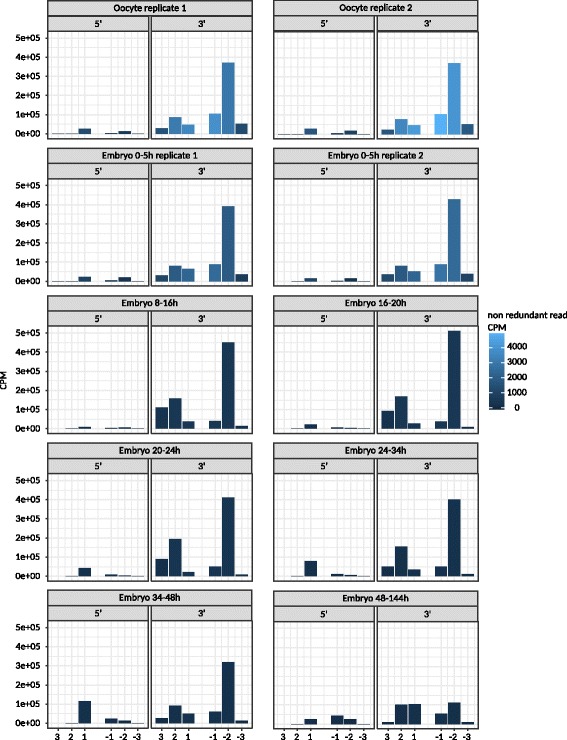
Templated 3' and 5' additions and deletions. The x-axis shows truncation in −1 steps and elongation in +1 steps and the y-axis shows the counts per million reads. The bar color displays the counts per million values of non-redundant reads supporting each miRNA variant

We next scanned for all non-templated nucleotide additions at the 3′ end. We confirmed that isomiRs with polyadenylate tails are strongly expressed in the oocyte and during the first embryonic stage; then expression weakens at the beginning of the first zygotic transcription phase (8 h). This reproduced the findings of the original study using the same dataset [15] (Additional file 1: Figure S4). Templated 3′ additions and deletions occurred very frequently in these datasets, although the expression level dropped below that of the unmodified mature microRNA in the final phase (48–144 h). In most cases, the 3′ end was shortened by two or three nucleotides compared to the original miRNA, but we also observed isomiRs that were elongated by two or three nucleotides during the 8–16 h and 24–34 h phases (Fig. 7). We observed a steady low level of 5′ isomiR expression with the exception of the penultimate and antepenultimate phases, where a single nucleotide 5′ extension was prevalent.

**Fig. 8:**
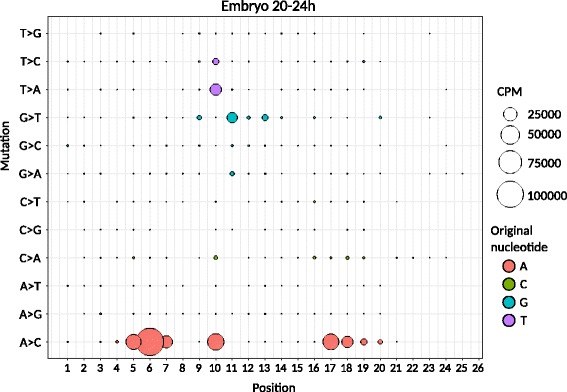
Detailed characterization of miRNA SNP expression in the embryo during the 20-24 h phase

During embryonic development, we observed a significant increase in the abundance of single-nucleotide mismatches during the 20–24 h stage, with a rapid decline immediately afterwards. We therefore characterized this phase in more detail, revealing frequent A-to-C mutations especially at position 5–7 in the microRNA seed region, and at positions 10 and 17–21 (Fig. 8). The latter segment lies directly behind the 3′ compensatory region (nucleotides 13–16) of the microRNA [6]. In addition, we observed an increase in T-to-C, T-to-A and G-to-T transitions before the compensatory region, spanning positions 10–13.

We observed an increase in the expression of mature microRNAs during the last four phases, including tca-miR-10-5p (Additional file 1: Figure S5). Furthermore, we observed an abrupt increase in the expression of tca-miR-376-3p, tca-bantam-3p and tca-miR-281-5p (among others) between the 34-48 h and 48-144 h phases. We observed an increase in the number of different mature miRNAs accumulating during each successive phase.

## Discussion

We evaluated the performance of three algorithms for the identification of isomiRs in small RNA sequencing data (isomiR-SEA, isomiRID and miraligner) and used the most suitable of the three (miraligner) to generate an overview of the isomiRome of the red flour beetle *Tribolium castaneum*. All three tools found it difficult to process technical errors, probably because we clustered the identical reads. This step reduced the number of correct reads to single copies, shrinking the majority of reads. All the unique mutations and mutations with few copies were also reduced to a non-redundant set. Therefore, only one copy of each original miRNA remained in the data along with multiple variants with one or more sequencing errors. This may have increased the number of false negatives because the missed sequences presumably lay outside the scope of the algorithms due to the higher error rate as expected from isomiRs. False negatives were therefore weighted as neutral for the scoring process. Although a sequencing error can mislead the results of the study, we considered is a benefit, when the tools were able to assign it. Later analysis may then filter out possible erroneous reads to improve the investigation results.

The evaluation of biological variants characterized the partially strong effects of sequence variations on the accuracy of isomiR identification. Both isomiRID and miraligner performed well, although miraligner was unable to identify all isomiRs with 3′ and 5′ deletions probably reflecting the seed-based search method. In contrast, isomiR-SEA performed poorly when mapping 5′ deletions and seed-mutated isoforms, but this was expected because the algorithm uses seed-based clustering for every miRNA and builds its entire analysis on these sets.

Each of the algorithms demonstrated particular strengths for specific applications. Although isomiR-SEA achieved the weakest overall evaluation score, it is likely to be the most promising tool to screen for diverse and highly mutated isomiRs because it is the only software that supports more than one mismatch. It is also the only tool that uses just the read sequences and a single sequence file with all already known mature microRNAs. This makes it ideal for non-model organisms, especially compared to isomiRID, which requires a genome file in addition to the files from miRBase. We assume that the visual output of isomiRID is designed for the manual evaluation of a small set of microRNAs. Because it is based on the bowtie1 aligner, it can only report one type of isoform per read and will not recognize combined mutations such as a mismatch combined with a templated 3′ addition. This can be checked visually but such combinations are not easily parsed by a pipeline. Finally, miraligner offered the best features of the other algorithms. It had a structured output comparable to isomiR-SEA, and scored nearly as much as isomiRID in terms of performance. It also makes use of miRBase files, but does not need a genome reference like isomiRID.

Having evaluated and compared all three algorithms, we then used miraligner to characterize the *T. castaneum* isomiRome during embryonic development. Our analysis revealed that the isomiRome is more diverse and dynamic than previously reported. We were able to reproduce earlier reports that polyadenylated miRNAs are expressed in the oocyte and during the first embryonic phase. We found that the number of isomiRs with 5′ extensions increases during the 24–34 h and 34–48 h phases, which may cause a seed shift in the miRNAs and therefore modify the range of mRNA targets. We also observed a high mutation rate within the seed region during the 20–24 h phase which would also have a strong effect on the range of mRNA targets. Many miRNAs showed a surge in expression during the last four phases, suggesting a greater need for those miRNAs before hatching. Those observations would now need to be investigated by target verification methods such as cross-linking immunoprecipitation.

## Conclusions

We evaluated the isomiR detection algorithms isomiR-SEA, isomiRID and miraligner, which are freely available and suitable for integration with local pipelines. We found that each program has advantages and disadvantages. Although isomiRID achieved the best performance against our evaluation criteria, the detailed visual output is more suitable for smaller datasets or the selected analysis of a few miRNAs. In contrast, isomiR-SEA gained a low score overall, but it allows the analysis of diverse mutations in large datasets because it accounts for more than one mutation in each miRNA, and because it can be run with only one file of mature miRNAs it is ideal for non-model organisms. Finally, we selected miraligner because it achieved a high-performance score and its clear output is ideal for pipeline integration. We used miraligner to screen the publicly available small RNA dataset of early development stages from *T. castaneum*, revealing the dynamic expression of isomiRs at each phase. These isomiRs must now be investigated in more detail to determine their biological functions.

## Additional file

Additional file 1: Supplemental figures. **Figure S1.** Analysis scheme for artificial test set evaluation. **Figure S2.** Pearson correlation of the length against the true positive, false positive and false negative rate. IsomiRID has a weak anti-correlation of length and false positive rate. Miraligner has a moderate anti-correlation of length and false negative rate. IsomiR-SEA has in both variations a weak anti-correlation of length and false negative rate. **Figure S3.** Detail view on the various lengths and their individual TP, FP and FN rates. **Figure S4.** Non-templated 3′ additions over all conditions. Strong expression of isomiRs with polyadenylate tails was observed in the oocyte and during the first embryonic phase. **Figure S5.** Expression of mature miRNAs during the last four embryonic phases. The number of mature miRNAs increases between the 20–24 h and 48–144 h phases. (DOCX 2207 kb)

## Abbreviations

isomiR: MicroRNA isoform
miRNA: MicroRNA
NGS: Next-generation sequencing
RISC: RNA-induced silencing complex
SNP: Single-nucleotide polymorphism
UTR: Untranslated region
