# Supplementary material for: Evaluation of high-throughput isomiR identification tools: illuminating the early isomiRome of Tribolium castaneum
Source: BMC Bioinformatics. 2017 Aug 3;18:359. doi: 10.1186/s12859-017-1772-z (PMC5543545; doi:10.1186/s12859-017-1772-z)
Supplement: Additional file 1: — Supplemental figures. Figure S1. Analysis scheme for artificial test set evaluation. Figure S2. Pearson correlation of the length against the true positive, false positive and false negative rate. IsomiRID has a weak anti-correlation of length and false positive rate. Miraligner has a moderate anti-correlation of length and false negative rate. IsomiR-SEA has in both variations a weak anti-correlation of length and false negative rate. Figure S3. Detail view on the various lengths and their individual TP, FP and FN rates. Figure S4. Non-templated 3′ additions over all conditions. Strong expression of isomiRs with polyadenylate tails was observed in the oocyte and during the first embryonic phase. Figure S5. Expression of mature miRNAs during the last four embryonic phases. The number of mature miRNAs increases between the 20–24 h and 48–144 h phases. (DOCX 2207 kb) [file 12859_2017_1772_MOESM1_ESM.docx]

Figure S1 **Analysis scheme for artificial test set evaluation**

Figure S2 **Pearson correlation of the length against the true positive, false positive and false negative rate.** IsomiRID has a weak anti-correlation of length and false positive rate. Miraligner has a moderate anti-correlation of length and false negative rate. IsomiR-SEA has in both variations a weak anti-correlation of length and false negative rate.

Figure S3 **Detail view on the various lengths and their individual TP, FP and FN rates**


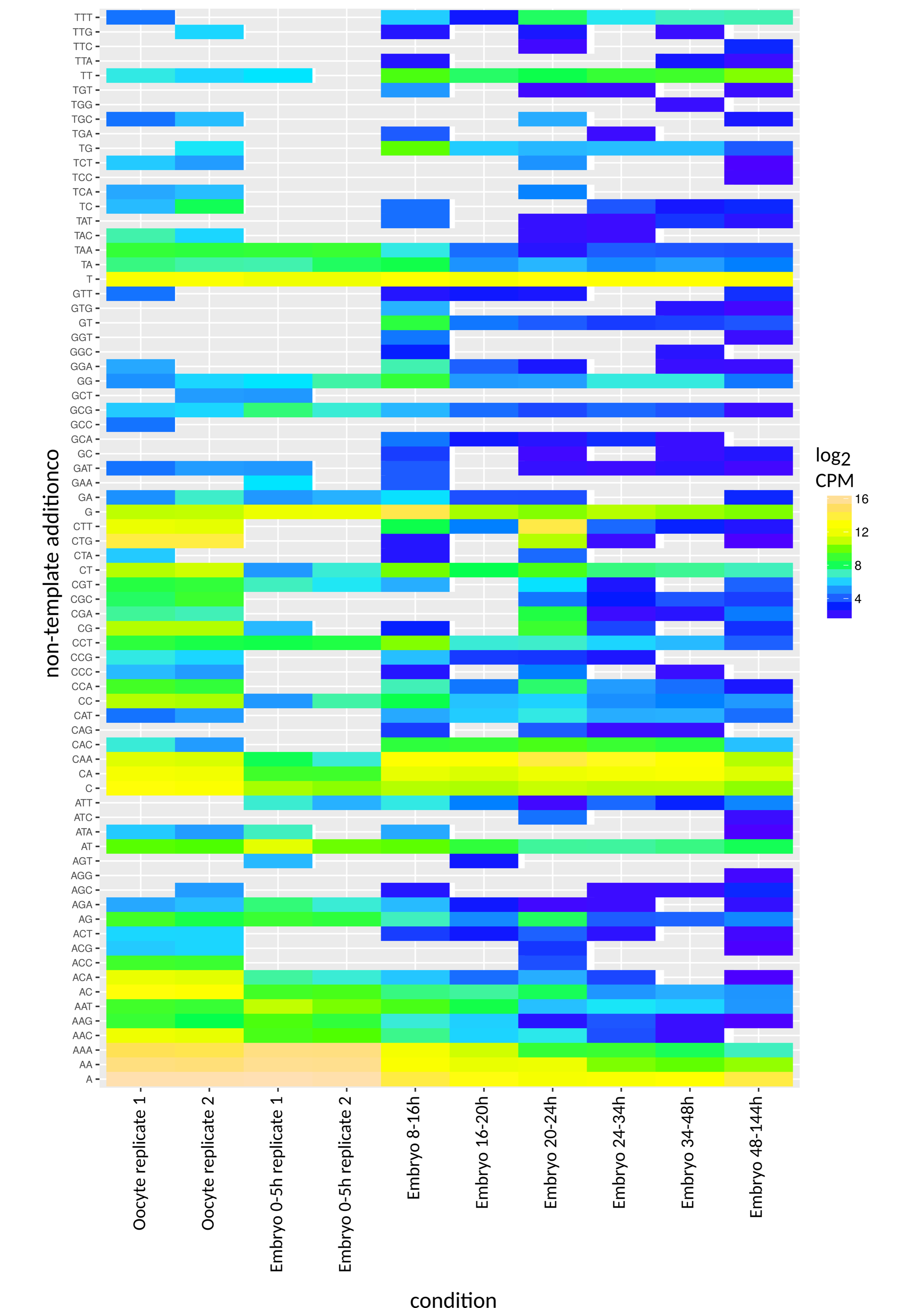


Figure S4 **Non-templated 3' additions over all conditions.** Strong expression of isomiRs with polyadenylate tails was observed in the oocyte and during the first embryonic phase.


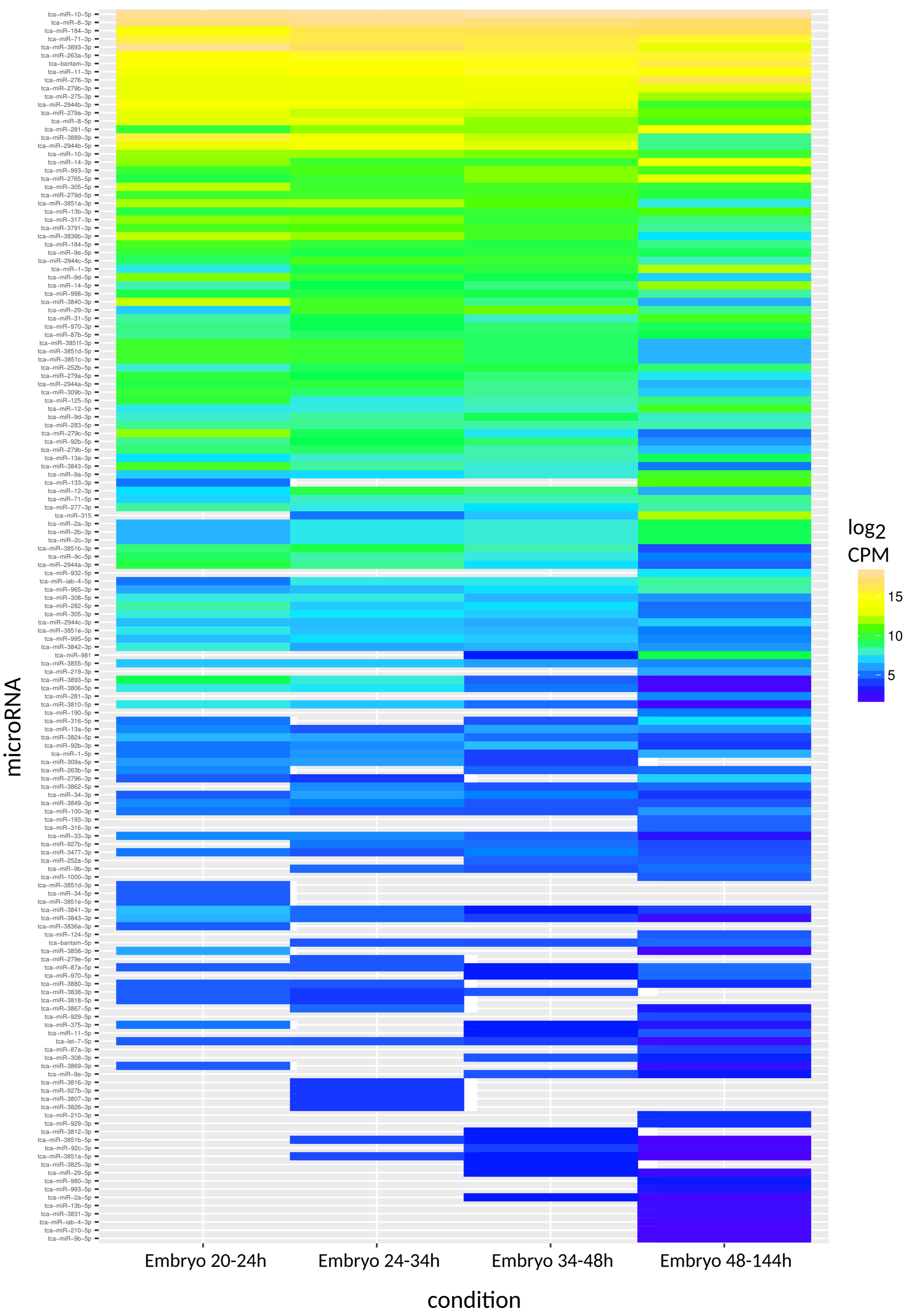


Figure S5 **Expression of mature miRNAs during the last four embryonic phases.** The number of mature miRNAs increases between the 20–24h and 48–144h phases.
